# Epistemic compression in large language model explanations of the gut–liver axis

**DOI:** 10.3389/fcimb.2026.1773593

**Published:** 2026-02-13

**Authors:** Man Sun, Dan Zang, Huan Zhou, Yi-Lin Che, Jun Chen

**Affiliations:** Department of Oncology, The Second Hospital of Dalian Medical University, Dalian, Liaoning, China

**Keywords:** epistemic compression, gut–liver axis, host–microbe interaction, informational reliability, intestinal microbiome, large language models

## Abstract

**Background:**

The gut–liver axis integrates intestinal barrier function, microbial ecology, metabolism, immune regulation, and hepatic feedback, yet remains causally non-closed and strongly context dependent. As large language models (LLMs) increasingly mediate biomedical explanation, their ability to preserve evidentiary structure within such epistemically open frameworks requires systematic evaluation.

**Methods:**

We conducted a cross-platform, mixed-methods infodemiology analysis of five widely accessible LLMs. Twenty clinically grounded questions spanning five hierarchical domains from basic mechanisms to intervention and evaluation generated 100 single-turn responses. Linguistic accessibility was assessed using seven established readability indices, while epistemic integrity was evaluated using the Journal of the American Medical Association Benchmark Criteria, Global Quality Score, and a modified DISCERN framework.

**Results:**

Linguistic complexity increased as prompts progressed toward intervention and evaluation, without corresponding gains in transparency, reliability, or educational quality. Informational integrity clustered primarily by platform rather than domain. Readability indices showed strong internal concordance, whereas integrity metrics aligned only moderately and correlated weakly with readability. Item-level analysis revealed consistently high narrative clarity but systematic under-signaling of source attribution and uncertainty, resulting in over-coherent explanations that compressed conditional associations into mechanism-like claims.

**Conclusions:**

LLM explanations of the gut–liver axis are susceptible to epistemic compression driven by narrative fluency rather than factual error. Readability does not reliably indicate epistemic robustness in decision-adjacent contexts. These findings support shifting evaluation and governance from platform comparison toward concept-conditioned requirement engineering that enforces provenance, calibrated uncertainty, and explicit separation of correlation, mechanism, and actionability as generative outputs approach clinical relevance.

## 1 Introduction

The gut–liver axis has emerged as a foundational systems framework in hepatology, describing how intestinal barrier physiology, microbial ecology, diet-derived metabolites, immune regulation, and hepatic feedback jointly shape homeostasis and disease progression (Albillos et al., 2020; Tilg et al., 2022; Pabst et al., 2023). Rather than a single linear pathway, it represents a barrier-and-circuit architecture spanning epithelial and vascular interfaces, bile acid recirculation, portal exposure, and immune imprinting (Peterson and Artis, 2014; Allaire et al., 2018). Its explanatory appeal lies in this integrative capacity across biological scales. Yet the framework remains causally non-closed. Evidence is strongly context dependent, varies across disease etiologies and stages, relies on surrogate markers of permeability and microbial function, and faces persistent challenges in causal attribution in human studies (Dreyer, 2022; Haedge et al., 2025). As a result, the scientific value of the gut–liver axis derives less from definitive mechanistic closure than from its ability to organize conditional and incomplete evidence within a coherent conceptual scaffold.

This epistemic structure has direct translational implications. Gut–liver axis reasoning is increasingly invoked to motivate diagnostic strategies, patient stratification, and intervention hypotheses across metabolic, inflammatory, and cholestatic liver diseases (Yan et al., 2023). At the same time, authoritative syntheses consistently caution that association-to-mechanism transitions remain uneven, that barrier dysfunction may be intermittent rather than persistent, and that diet, medication exposure, immune state, and host context substantially complicate inference and trial design (Lowe, 2018). The explanatory utility of the gut–liver axis therefore depends on preserving evidentiary gradients, clearly distinguishing correlation from plausible mechanism and mechanism from clinical actionability (Hsu and Schnabl, 2023; Li et al., 2025). Any explanatory system that collapses these distinctions risks overstating evidentiary maturity precisely where clinical interpretation becomes most consequential.

Large language models (LLMs) are rapidly becoming default interfaces for biomedical explanation in patient-facing and decision-adjacent settings (Yu et al., 2024; Cao et al., 2025; Lobentanzer et al., 2025). Their strengths in fluent synthesis, abstraction, and narrative integration align closely with the integrative logic of the gut–liver axis, which naturally invites cross-scale bridging between microbiology, metabolism, immunology, and clinical phenotype. In epistemically open frameworks, however, this alignment introduces a predictable distortion. Conditional evidence can be rendered as settled mechanism, and uncertainty absorbed into rhetorical completeness rather than explicitly articulated. We refer to this structural failure mode as epistemic compression, whereby fluent generative narration transforms conditional associations into mechanism-like explanations, particularly as prompts approach intervention and evaluation. In this context, the dominant risk is not isolated factual error, but premature closure driven by narrative coherence.

Existing evaluations of LLM health content have largely assessed accuracy, hallucination, or readability in isolation, often treating linguistic simplicity as a proxy for safety (Asgari et al., 2025; Bedi et al., 2025). This assumption is fragile for multi-system biomedical frameworks in which the principal hazard is erosion of evidentiary boundaries rather than overt misinformation (Piquer-Martinez et al., 2024). Here, we conduct a cross-platform, mixed-methods infodemiology analysis of how widely accessible LLMs explain the gut–liver axis, jointly profiling linguistic accessibility and epistemic integrity across a hierarchy from basic mechanisms to decision-adjacent use. By applying established instruments for transparency and reliability alongside multi-metric readability assessment, we aim to identify structural failure modes that conventional benchmarking does not capture. Treating model outputs as engineered epistemic artifacts rather than neutral summaries, this study seeks to derive concept-conditioned governance principles for the safe deployment of generative systems in translational bioengineering and clinical contexts (Knuuttila, 2021).

## 2 Methods

### 2.1 Overall study framework

We conducted a cross-sectional, mixed-methods infodemiology study to systematically benchmark how LLMs explain the gut–liver axis, a biologically and clinically integrative concept spanning microbiology, metabolism, immunology, and clinical medicine. Rather than quantifying factual accuracy or hallucination frequency, this study focused on the structural properties of explanation that shape evidentiary signaling, an aspect critical for decision-adjacent applications. The evaluation was explicitly centered on two complementary dimensions with direct relevance to translational bioengineering and clinical decision support: informational reliability and patient-facing interpretability. The study design was constructed to approximate real-world information-seeking behavior while maintaining strict experimental control, thereby enabling reproducible, model-agnostic comparisons across platforms. As the analysis was restricted to model-generated text and did not involve human participants, clinical records, or biological specimens, institutional ethics review and informed consent were not required.

To probe model performance across increasing levels of mechanistic and clinical complexity, we developed a structured set of 20 clinically grounded questions through an iterative, expert-led process. Three oncology physicians with active roles in patient education and clinical decision-making integrated contemporary clinical guidelines, validated patient-education resources, and recurrent informational needs observed in routine outpatient practice to define representative queries (**Table 1**). Questions were refined to ensure neutral phrasing, conceptual precision, and patient-centered relevance, and were subsequently organized into five domains reflecting the hierarchical knowledge structure of the gut–liver axis: basic mechanism, microbiota–metabolism, disease association, clinical intervention, and detection and evaluation. This domain-based framework was designed to identify points at which fluent narrative generation diverges from mechanistic fidelity, a known limitation when LLM outputs are used in decision-adjacent contexts.

**Table 1:** Issue list.

Issue list
I. Basic mechanism dimension
1. How do the anatomical and molecular signaling pathways of the gut-liver axis cooperate?
2. Mechanism by which gut microbiota metabolites regulate liver function via gut-liver axis?
3. Core role of intestinal mucosal barrier integrity in maintaining gut-liver axis homeostasis?
4. How does enterohepatic circulation of bile acids mediate gut-liver bidirectional communication?
II. Microbiota-metabolism dimension
1. Principle of probiotics regulating gut-liver axis balance to improve metabolic-related liver diseases?
2. Mechanism by which gut microbiota dysbiosis induces non-alcoholic fatty liver via gut-liver axis?
3. How do microbiota metabolites like short-chain fatty acids affect liver immunity via gut-liver axis?
4. How does dietary structure reshape gut microbiota and liver function through gut-liver axis?
III. Disease association dimension
1. Key role of gut-liver axis dysfunction in the development and progression of liver cirrhosis?
2. Pathological mechanism of inflammatory bowel disease causing secondary liver injury via gut-liver axis?
3. Correlation study between gut-liver axis imbalance and primary biliary cholangitis?
4. Molecular mechanism of gut-liver axis barrier injury in patients with alcoholic liver disease?
IV. Clinical intervention dimension
1. Application value of gut-liver axis-targeted probiotic therapy in liver disease treatment?
2. Clinical evidence of fecal microbiota transplantation improving liver diseases via gut-liver axis?
3. Adjuvant therapeutic effect of dietary fiber supplementation on gut-liver axis-related liver diseases?
4. Current progress and clinical translation challenges of gut-liver axis-targeted drug development?
V. Detection and evaluation dimension
1. Combined strategy of serological and fecal biomarkers for gut-liver axis function evaluation?
2. Application of non-invasive imaging techniques in diagnosing gut-liver axis-related diseases?
3. Evaluating gut-liver axis homeostasis by gut microbiota sequencing combined with liver function indicators?
4. Construction and validation method of clinical scoring system for gut-liver axis dysfunction?

All questions were submitted verbatim to five widely accessible LLMs within a predefined collection window (December 10–20, 2025) to minimize version drift and enhance reproducibility. Each query was entered in a new, independent session without supplemental instructions, contextual priming, or follow-up prompts (Al-Thani et al., 2023). When multiple candidate responses were generated, the first complete output was retained to limit investigator degrees of freedom. Outputs were compiled into a standardized, anonymized corpus of 100 responses indexed by model and question domain, forming the basis for subsequent quantitative scoring and qualitative error-pattern analysis. The fixed sample size of 20 responses per platform was selected to enable controlled cross-platform and cross-domain comparison under standardized prompting conditions, prioritizing internal validity and pattern detection over statistical power for population-level inference regarding overall model performance. This framework was designed to function as a structured audit of explanatory performance rather than a benchmark of stylistic fluency.

### 2.2 Assessment of readability

To characterize the ease with which LLM-generated outputs can be processed by a lay audience, we assessed linguistic accessibility using a multi-metric readability framework. This analysis was intended to describe surface-level language burden rather than infer scientific validity, thereby explicitly separating textual form from informational substance.

Seven established readability indices were applied to each response (**Supplementary Table 1**), capturing complementary dimensions of linguistic complexity, including syntactic structure, lexical length, and sentence density: Coleman–Liau Score (CL), Linsear Write Index (LW), Automated Readability Index (ARI), Simple Measure of Gobbledygook (SMOG), Gunning Fog Index (GFOG), Flesch Reading Ease Score (FRES), and Flesch–Kincaid Grade Level (FKGL) (Mac et al., 2022; Özduran and Hanci, 2022; Yilmaz Hanci, 2023; Hanci et al., 2024). With the exception of FRES, higher values indicate greater textual complexity.

All calculations were performed on raw, unedited model outputs using uniform computational settings across models. This approach preserved intrinsic language characteristics and ensured that readability comparisons reflected model behavior rather than post-processing artifacts. Readability metrics were treated as descriptive indicators of user-facing communication complexity and were evaluated independently from content-level quality to avoid conflating accessibility with epistemic validity (Ojha et al., 2021; Gkikas et al., 2022). Accordingly, readability metrics were treated as descriptive indicators of linguistic complexity and user-facing communication burden, rather than as normative proxies for informational quality, accuracy, or epistemic reliability.

### 2.3 Assessment of informational reliability and quality

Beyond linguistic form, we independently examined the epistemic quality of model-generated explanations, focusing on transparency, reliability, and educational value. Informational integrity was operationalized using three complementary instruments with established use in digital health information assessment: the Journal of the American Medical Association (JAMA) Benchmark Criteria, the Global Quality Score (GQS), and a modified DISCERN framework (**Supplementary Table 2**) (Charnock et al., 1999; Azer, 2020; Kyarunts et al., 2022; Mohammadzadeh Azarabadi et al., 2025).

Transparency and source signaling were assessed using the JAMA Benchmark Criteria, which capture explicit disclosure of authorship, attribution of information sources, reporting of conflicts or sponsorship, and temporal currency (Bruce-Brand et al., 2013; Kara et al., 2024). Each criterion was scored dichotomously, yielding a composite score ranging from 0 to 4.

Educational value was evaluated using the GQS, a five-point ordinal scale reflecting overall coherence, logical organization, and potential usefulness for non-specialist audiences(Uzun, n.d.). Higher scores indicate content judged to be clearer and more informative from a patient-education perspective.

To further assess internal content reliability, we applied a modified DISCERN instrument restricted to its first domain, consistent with prior evaluations of non-interactive health information (Handler et al., 2021; Shan et al., 2023). The full DISCERN instrument was not applied because its subsequent domains primarily assess treatment options and decision-making quality, which are less applicable to non-interactive, explanatory model outputs rather than patient-facing treatment guidance. This domain comprises five binary items addressing clarity of purpose, relevance and balance, identification of sources, and acknowledgment of uncertainty. Affirmative responses were summed to generate a reliability score ranging from 0 to 5.

### 2.4 Scoring consistency and reviewer agreement

To mitigate subjectivity in content-level judgments, all responses underwent independent dual evaluation by medically trained reviewers following a structured calibration procedure. Prior to formal scoring, reviewers jointly assessed a set of pilot responses to harmonize interpretation of scoring criteria across all instruments. aInter-reviewer agreement was quantified using Cohen’s kappa statistic to measure consistency beyond chance, interpreted according to established benchmarks (McHugh, 2012; Rau and Shih, 2021). Discrepancies were resolved through structured discussion, with adjudication by a senior reviewer when necessary. This process ensured that observed differences in quality metrics reflected genuine variation in model outputs rather than inconsistency in evaluator judgment.

### 2.5 Statistical analysis

All analyses followed a prespecified workflow to ensure reproducible, model-agnostic inference while accounting for corpus hierarchy. Normality was assessed using the Shapiro–Wilk test and visual inspection. Between-model differences were evaluated using one-way analysis of variance with Bonferroni adjustment for approximately normal measures, and Kruskal–Wallis tests with Dunn–Bonferroni correction for non-normal measures, including most readability indices. Standardized effect sizes with confidence intervals were reported alongside P values. Associations between readability and informational reliability and quality were analyzed using Spearman correlations and domain-adjusted partial correlations. All tests were two-sided with P < 0.05. Statistical analyses were conducted in IBM SPSS Statistics (version 25), and figures were generated using GraphPad Prism (version 9).

## 3 Results

### 3.1 Readability and informational quality show distinct domain- and platform-level patterns

A total of 100 LLM-generated responses were analyzed, evenly distributed across five predefined gut–liver axis knowledge domains. Linguistic accessibility varied systematically by domain, with statistically significant heterogeneity concentrated in indices reflecting grade-level demand and syntactic density (**Table 2**). ARI, GFOG, FKGL, and CL all demonstrated higher median values in domains oriented toward disease association, clinical intervention, and evaluation compared with foundational mechanistic domains, delineating a domain-dependent complexity gradient.

**Table 2:** Readability and informational quality metrics across gut–liver axis knowledge domains.

Variables	Total (n = 100)	Basic mechanism dimension (n = 20)	Clinical intervention dimension (n = 20)	Detection and evaluation dimension (n = 20)	Disease association dimension (n = 20)	Microbiota-metabolism dimension (n = 20)	Statistic	*P*
M Dis Score, Mean ± SD	2.80 ± 1.20	2.80 ± 1.54	2.65 ± 1.09	2.85 ± 1.14	3.00 ± 1.17	2.70 ± 1.08	F=0.25	0.907
JAMA score, Mean ± SD	2.19 ± 1.09	2.20 ± 1.20	2.30 ± 1.13	1.90 ± 1.07	2.10 ± 1.12	2.45 ± 0.94	F=0.72	0.582
GQS score, Mean ± SD	2.79 ± 1.14	2.45 ± 1.19	2.65 ± 1.14	2.90 ± 0.97	3.30 ± 1.22	2.65 ± 1.09	F=1.69	0.159
ARI, M (Q_1_, Q_3_)	19.81 (18.26, 22.15)	18.98 (17.84, 19.95)	19.50 (18.73, 20.51)	22.20 (19.23, 25.91)	20.88 (19.13, 23.15)	19.84 (17.97, 24.30)	χ²=11.66#	0.020
FRES, M (Q_1_, Q_3_)	0.00 (0.00, 4.00)	2.50 (0.00, 10.00)	0.00 (0.00, 1.00)	0.00 (0.00, 0.00)	0.00 (0.00, 1.50)	0.00 (0.00, 2.25)	χ²=5.86#	0.210
GFOG, M (Q_1_, Q_3_)	20.40 (18.60, 22.63)	18.90 (18.08, 20.75)	19.20 (17.70, 20.40)	21.00 (19.70, 23.75)	22.05 (20.38, 24.20)	21.05 (18.78, 22.88)	χ²=14.77#	0.005
FKGL, M (Q_1_, Q_3_)	18.59 (17.03, 20.82)	17.98 (16.97, 18.34)	18.34 (17.03, 19.53)	20.75 (18.38, 23.18)	20.09 (17.92, 21.60)	18.28 (16.65, 22.29)	χ²=10.93#	0.027
CL, M (Q_1_, Q_3_)	21.62 (19.34, 23.84)	19.50 (18.37, 21.26)	20.85 (19.60, 22.18)	23.52 (20.24, 24.44)	23.22 (20.72, 25.05)	22.14 (19.97, 23.67)	χ²=13.72#	0.008
SMOG, M (Q_1_, Q_3_)	15.01 (14.11, 16.61)	14.77 (13.89, 15.44)	14.96 (13.82, 15.90)	17.24 (14.36, 18.52)	15.39 (14.43, 16.83)	14.64 (14.31, 17.61)	χ²=9.28#	0.055
LW, M (Q_1_, Q_3_)	50.00 (45.00, 53.00)	51.00 (47.50, 53.00)	50.00 (47.00, 54.25)	48.00 (39.00, 51.25)	48.50 (43.75, 52.00)	50.00 (45.50, 54.25)	χ²=5.27#	0.261

F, ANOVA; #, Kruskal–Wallis test; SD, standard deviation; M, Median; Q_1_, 1st Quartile; Q_3_, 3rd Quartile. Red values indicate statistically significant differences (P < 0.05).

In contrast, surface-level fluency measures showed limited sensitivity to domain context, and no domain-dependent differences were observed for informational reliability or educational quality indicators, including the modified DISCERN score, JAMA Benchmark score, and Global Quality Score (all P > 0.05). Together, these findings indicate that increasing linguistic complexity across the gut–liver axis knowledge hierarchy is not accompanied by parallel gains in epistemic robustness. As a result, topic complexity alone cannot be used to infer informational quality, indicating that greater interpretive demands are not accompanied by corresponding gains in informational reliability.

### 3.2 Platform identity is the primary determinant of informational reliability and quality

**Figure 1:**
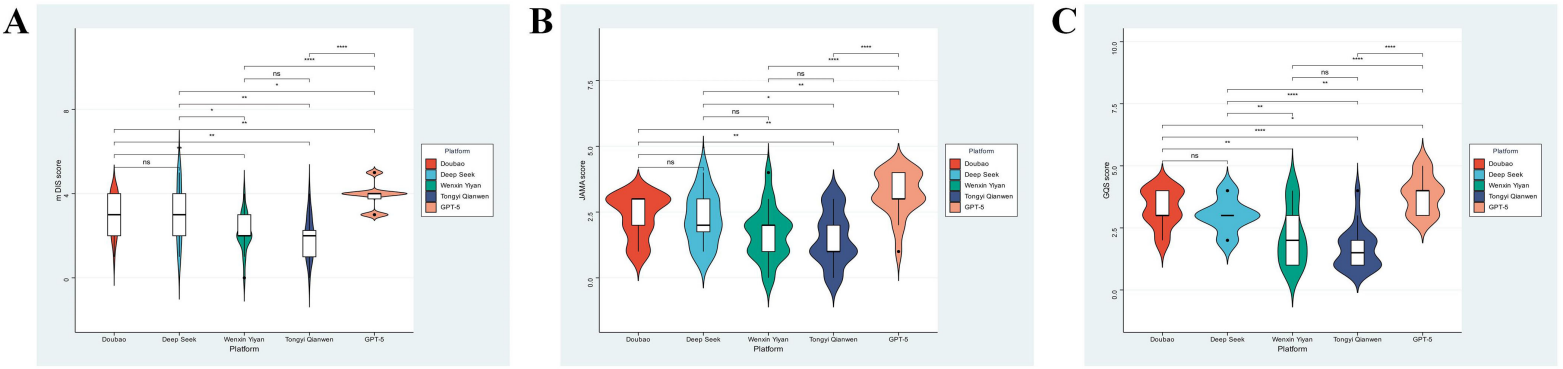
Distribution of informational quality scores across large language model platforms. Violin plots display the distribution of modified DISCERN scores **(A)**, JAMA Benchmark scores **(B)**, and Global Quality Scores **(C)** for gut–liver axis explanations generated by five large language models (n = 20 responses per platform). Central lines indicate medians and shaded areas represent interquartile ranges.

Across all evaluated responses, platform identity accounted for the largest share of variation in informational reliability and educational quality (**Figures 1A–C**; **Table 3**). Statistically significant between-model differences were observed for the modified DISCERN score, JAMA Benchmark Criteria, and Global Quality Score (all P < 0.001), demonstrating that explanatory performance clustered systematically by platform rather than being evenly distributed across outputs. These cross-platform differences are not intended to constitute a performance ranking or hierarchy of model superiority, but rather to illustrate distinct explanatory strategies and epistemic signaling patterns across models under standardized conditions. GPT-5 consistently achieved the highest mean scores across all three instruments, indicating more robust transparency signaling, stronger reliability features, and greater educational utility, whereas Tongyi Qianwen ranked lowest overall, reflecting limited robustness in delivering trustworthy, decision-relevant explanations of the gut–liver axis. Other platforms occupied intermediate positions with broader score dispersion, suggesting greater variability in output-level reliability.

**Table 3:** Readability and informational quality metrics across LLM platforms.

Variables	Total (n = 100)	Deep seek (n = 20)	Doubao (n = 20)	GPT-5 (n = 20)	Tongyi Qianwen (n = 20)	Wenxin Yiyan (n = 20)	Statistic	*P*
M Dis Score, Mean ± SD	2.80 ± 1.20	3.05 ± 1.36	3.00 ± 0.92	3.90 ± 0.64	1.90 ± 0.97	2.15 ± 0.88	F=13.27	<0.001
JAMA score, Mean ± SD	2.19 ± 1.09	2.25 ± 0.97	2.35 ± 0.81	3.20 ± 0.83	1.50 ± 1.00	1.65 ± 0.99	F=10.65	<0.001
GQS score, Mean ± SD	2.79 ± 1.14	3.00 ± 0.65	3.30 ± 0.73	3.85 ± 0.75	1.65 ± 0.81	2.15 ± 1.14	F=22.63	<0.001
ARI, M (Q_1_, Q_3_)	19.81 (18.26, 22.15)	20.78 (18.88, 27.33)	22.48 (20.73, 25.00)	19.49 (18.87, 19.92)	20.29 (18.86, 21.52)	17.26 (16.08, 18.17)	χ²=43.47#	<0.001
FRES, M (Q_1_, Q_3_)	0.00 (0.00, 4.00)	0.00 (0.00, 4.50)	0.00 (0.00, 0.00)	0.00 (0.00, 0.00)	0.00 (0.00, 0.25)	10.00 (1.75, 21.25)	χ²=37.78#	<0.001
GFOG, M (Q_1_, Q_3_)	20.40 (18.60, 22.63)	20.85 (18.78, 23.75)	22.85 (20.20, 24.27)	20.80 (20.23, 21.92)	21.00 (18.50, 21.78)	17.65 (16.45, 19.20)	χ²=31.20#	<0.001
FKGL, M (Q_1_, Q_3_)	18.59 (17.03, 20.82)	20.06 (17.41, 24.50)	20.79 (19.65, 22.37)	18.41 (17.74, 19.09)	18.50 (17.03, 20.97)	16.61 (15.26, 17.67)	χ²=31.81#	<0.001
CL, M (Q_1_, Q_3_)	21.62 (19.34, 23.84)	22.63 (19.61, 24.86)	23.28 (21.06, 24.50)	23.34 (22.36, 24.03)	20.94 (20.03, 22.50)	17.39 (16.17, 18.94)	χ²=45.21#	<0.001
SMOG, M (Q_1_, Q_3_)	15.01 (14.11, 16.61)	16.00 (14.84, 19.66)	17.34 (16.06, 18.21)	14.11 (13.82, 14.42)	15.01 (14.71, 16.51)	14.21 (13.28, 15.07)	χ²=43.22#	<0.001
LW, M (Q_1_, Q_3_)	50.00 (45.00, 53.00)	46.50 (39.75, 51.25)	43.50 (40.50, 47.25)	52.00 (50.00, 53.00)	50.50 (48.50, 53.00)	52.00 (47.75, 55.00)	χ²=31.14#	<0.001

F, ANOVA; #, Kruskal–Wallis test; SD, standard deviation; M, Median; Q_1_, 1st Quartile; Q_3_, 3rd Quartile. Red values indicate statistically significant differences (P < 0.05).

Readability likewise varied significantly by platform across all assessed indices, including ARI, FRES, GFOG, FKGL, CL, SMOG, and LW (all P < 0.001). Some platforms consistently generated explanations with higher grade-level demand and greater syntactic density, whereas others produced linguistically simpler outputs. Importantly, lower linguistic complexity was not associated with improved informational reliability, and higher language demand was not intrinsically detrimental. Together, these results indicate that platform-level optimization of readability and epistemic reliability proceeds along partially independent trajectories, establishing platform identity as the dominant determinant of explanation quality in LLM-generated gut–liver axis content.

### 3.3 Readability does not predict informational reliability or quality across platforms

**Figure 2:**
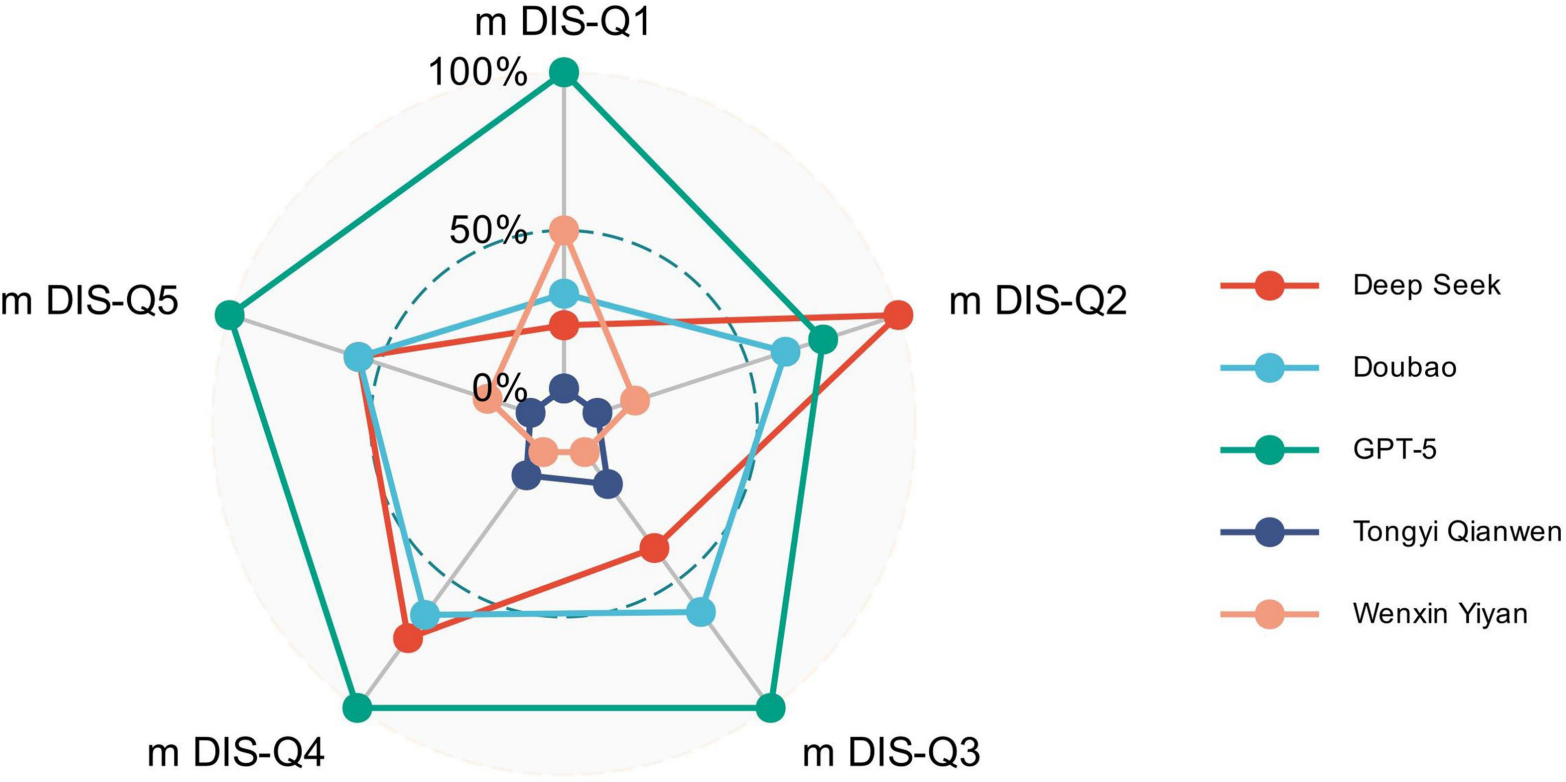
Item-level reliability profiles across large language model platforms based on the modified DISCERN framework. Radar plots illustrate the percentage of responses meeting each modified DISCERN criterion (mDIS-Q1 to mDIS-Q5) across five large language model platforms (n = 20 responses per platform). Each axis corresponds to one reliability component, allowing comparison of item-level reliability configurations across models.

Item-level analysis using the modified DISCERN framework showed that between-model differences were driven by reconfiguration of specific reliability components rather than uniform shifts in overall quality (**Figure 2**). Across platforms, attainment in presentation-oriented items, including clarity of purpose and informational balance, did not consistently coincide with attainment in credibility-signaling items, particularly source identification and acknowledgment of uncertainty. Consequently, composite reliability scores masked distinct internal configurations of epistemic strength and weakness, indicating that narrative adequacy can coexist with systematic gaps in evidentiary accountability.

Distinct reliability profiles emerged at the item level. GPT-5 demonstrated uniformly high attainment across all DISCERN items, reflecting consistent coupling of narrative clarity with source disclosure and uncertainty awareness. In contrast, other platforms exhibited selective or constrained patterns characterized by persistent underperformance in disclosure-oriented dimensions, resulting in attenuated composite reliability signals. These structured omission profiles underscore the value of item-level reliability assessment for identifying failure modes that are not captured by aggregate quality scores.

### 3.4 Platform-specific trade-offs define distinct explanatory profiles

**Figure 3:**
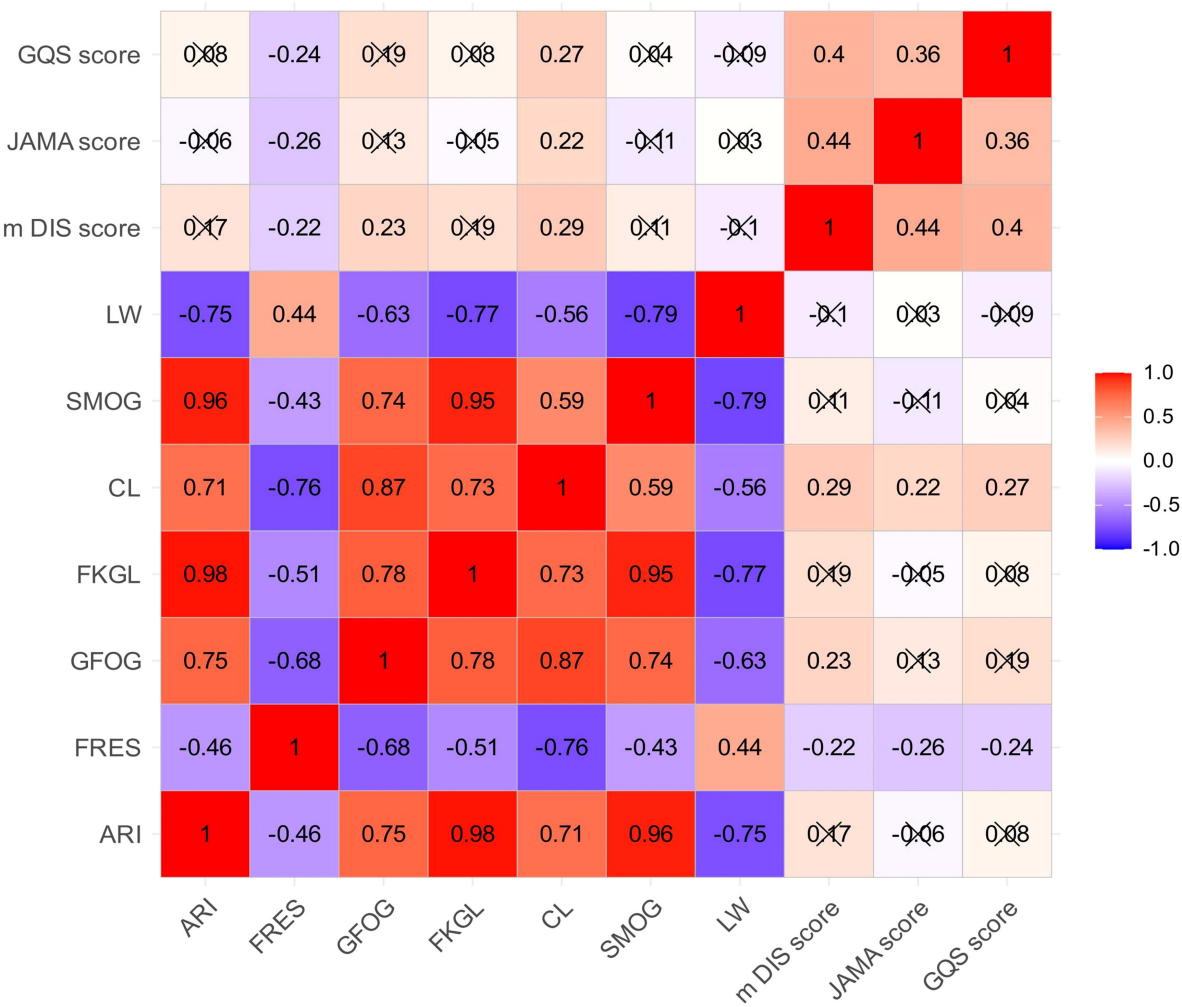
Correlation structure between readability indices and informational quality metrics. Heatmap displays pairwise Spearman correlation coefficients between readability indices and informational quality metrics, including the modified DISCERN score, JAMA Benchmark score, and Global Quality Score, for LLM-generated gut–liver axis explanations. Color intensity reflects the strength and direction of correlations.

Correlation analysis demonstrated that linguistic accessibility and epistemic reliability occupy largely separable dimensions in LLM-generated gut–liver axis explanations (**Figure 3**). Readability indices formed a tightly coupled cluster, with strong concordance among grade-level demand and syntactic density measures, whereas integrity-oriented scores showed only moderate convergence, indicating that transparency signaling, reliability cues, and perceived educational value are related but non-identical constructs.

Cross-dimensional associations between readability and informational integrity were uniformly weak. Modified DISCERN, JAMA Benchmark, and Global Quality Score each exhibited only small or near-null correlations with grade-level demand and fluency measures, demonstrating that linguistic simplicity cannot be used as a proxy for credibility or trustworthiness. Together, these findings define a structural partition between language burden and epistemic safeguards, indicating that readability and informational reliability represent independent axes of explanatory performance rather than interchangeable indicators of explanation quality.

## 4 Discussion

This study was not designed to rank LLMs, but to clarify why the gut–liver axis is particularly vulnerable to epistemic distortion when rendered as fluent generative explanation. Importantly, these findings should not be interpreted as a negation of the overall utility or technical potential of large language models in biomedical domains. Rather, they are intended to inform targeted safeguards that support safer and more reliable deployment of generative systems in decision-adjacent clinical and translational contexts. The axis is not a closed pathway but a coordination framework spanning intestinal barrier physiology, microbial ecology, diet-derived metabolites, immune regulation, and heterogeneous clinical phenotypes (Zhuang et al., 2024; O’Riordan et al., 2025; Park et al., 2025). Its explanatory appeal lies in this integrative capacity. Yet its evidentiary base remains conditional and context dependent, with substantial variation across disease etiologies and stages, reliance on imperfect surrogate markers of barrier function, and persistent challenges in causal attribution. In such a setting, narrative completeness is readily produced, whereas evidentiary closure is difficult to justify, creating a predictable tension for generative narration.

Within this context, the dominant risk observed was premature closure rather than isolated factual error. As prompts progressed from foundational mechanisms toward association, intervention, and evaluation, linguistic complexity and narrative integration increased, while indicators of informational reliability and educational value did not strengthen in parallel (Supriyono et al., 2024). Disclosure of provenance and explicit acknowledgment of uncertainty remained limited even as explanations became more elaborate and decision oriented (Djulbegovic, 2001; Wang et al., 2024). Epistemically open claims were thus reframed as rhetorically resolved accounts. This asymmetry is particularly consequential in the gut–liver axis literature, where associative findings are abundant but mechanistic validation and intervention-level evidence remain uneven (Szabo, 2015; Kim et al., 2024; Xing et al., 2025). When conditional relationships are compressed into coherent causal arcs, plausibility can be misinterpreted as settled knowledge, especially in decision-adjacent contexts (Correia et al., 2022).

Platform-level analyses further indicate that this distortion is shaped primarily by generation strategy rather than topic difficulty alone (Han et al., 2024). Accordingly, the observed platform-level differences should be interpreted as robust structural patterns within a controlled explanatory corpus, rather than as estimates of population-level or longitudinal model performance. Informational reliability and educational quality clustered by model, whereas domain effects were comparatively modest (Briceno-Rosas, 2025). Readability also varied substantially across platforms, yet reduced linguistic burden did not confer greater epistemic robustness, and higher grade-level demand was not intrinsically detrimental (Qiao and Feng, 2024). Item-level reliability profiling helps explain this pattern. Across platforms, presentation-oriented features such as purpose clarity and topical balance were more consistently achieved than accountability primitives, particularly source identification and uncertainty signaling. This imbalance mirrors a central bottleneck in gut–liver axis research itself, where correlations across microbiome composition, metabolites, and disease phenotypes often outpace causal validation (Chen et al., 2025). When provenance and uncertainty are under-signaled, associative links can be implicitly elevated into mechanistic or intervention-relevant claims, flattening the evidence gradient and obscuring distinctions between correlation, plausible mechanism, and clinical sufficiency.

From a translational bioengineering perspective, these findings argue for treating LLM outputs as engineered epistemic artifacts rather than neutral summaries. Explanations of the gut–liver axis inevitably encode assumptions about causality, evidence tier, and readiness for action, and fluent narration can exceed the maturity of the underlying science in the absence of explicit constraints (Mandato et al., 2021). Governance should therefore shift from model selection to requirement engineering. Minimum specifications should include explicit separation of associative, mechanistic, and intervention-relevant claims; clear signaling of evidence type or source class; calibrated expression of uncertainty reflecting context dependence; and boundary statements defining what an explanation cannot support, with progressively stricter constraints as prompts approach intervention and evaluation (Marchionni and Reijula, 2019). At the same time, future evaluation and design efforts should move beyond aggregate quality scores toward domain-aware integrity profiling that explicitly tests preservation of evidence gradients under decision pressure, and toward constraint-aware generation strategies in which uncertainty calibration and evidence-tier separation are embedded into the explanatory process rather than assessed *post hoc*. Such approaches would better align generative systems with the translational reality of gut–liver axis research, where associative findings frequently outpace causal validation, and would help prevent fluent accessibility from becoming a conduit for premature closure.

## 5 Conclusion

This cross-platform analysis demonstrates that LLMs explaining the gut–liver axis are vulnerable to systematic epistemic distortion driven by narrative fluency rather than factual error alone. Linguistic accessibility and epistemic integrity were consistently decoupled, indicating that readability cannot serve as a proxy for reliability in decision-adjacent biomedical communication. Over-coherent explanations frequently compressed conditional associations into mechanism-like narratives while under-signaling uncertainty and provenance. Together, these findings support a shift from model comparison toward concept-conditioned requirement engineering, with explicit safeguards to preserve evidentiary gradients as generative explanations approach clinical and translational use.

## Data Availability

The original contributions presented in the study are included in the article/**Supplementary Material**. Further inquiries can be directed to the corresponding author.
