# Supplementary material for: Epistemic compression in large language model explanations of the gut–liver axis
Source: Front Cell Infect Microbiol. 2026 Feb 13;16:1773593. doi: 10.3389/fcimb.2026.1773593 (PMC12945831; doi:10.3389/fcimb.2026.1773593)
Supplement: Supplementary file 1 [file Table1.docx]

Supplementary Table 1. Readability tools, formulas and descriptions.

| Readability Index | Description | Formula |
| --- | --- | --- |
| Gunning FOG (GFOG) | It estimates the number of years of education required for a person to understand a given text. | G=0.4 X (W/S+((C*W) X 100)) |
| Flesch Reading Ease Score (FRES) | It was created to assess the readability of newspapers and is particularly effective for evaluating school textbooks and technical manuals. The scores range from 0 to 100, with higher scores indicating greater ease of reading. | I = (206.835 – (84.6 X (B/W)) – (1.015 X (W/S))) |
| Flesch–Kincaid grade level (FKGL) | Delineates the academic capacity level imperative for grasping the written material | G = (11.8 X (B/W)) + (0.39 X (W/S)) – 15.59 |
| Simple Measure of Gobbledygook (SMOG) | It measures the number of years of education the average person needs to understand a text. | G=1.0430 X √C + 3.1291 |
| Coleman–Liau (CL) score | Evaluates the educational level required for understanding a text and offers an associated grade level in the US education system. | G = (–27.4004 X (E/100)) + 23.06395 |
| Linsear Write (LW) | Offers an approximate assessment of the academic level needed to comprehend the text. | LW = (R+3C)/S Result   • If >20, divide by 2   •If ≤20, subtract 2, and then divide by 2 |
| Automated readability index (ARI) | Assesses the scholastic rank in American educational institutions needed to be capable of comprehending written material. The greater the number of characters, the more complex the term. | ARI=4.71 X I+0.5*ASL – 21.43 |

Note: G=Grade level; B=Number of syllables; W=Number of words; S=Number of sentences; I=Flesch Index Score; SMOG=Simple Measure of Gobbledygook; C=Complex words (≥3 syllables); E=predicted Cloze percentage=141.8401 – (0.214590 X number of characters) + (1.079812*S); C*=Complex words with exceptions including, proper nouns, words made 3 syllables by addition of “ed” or “es”, compound words made of simpler words. ASL=the average number of sentences per 100 words R=the number of words ≤2 syllables.
